# Rumination as a Mediator of the Prospective Association Between Victimization and Bullying

**DOI:** 10.1007/s10802-020-00755-z

**Published:** 2021-01-06

**Authors:** Sarah T. Malamut, Christina Salmivalli

**Affiliations:** Department of Psychology, INVEST Research Flagship, University of Turku, 20500 Turku, Finland

**Keywords:** Victimization, Bullying, Rumination, Anger

## Abstract

Although there is evidence of concurrent associations between victimization and bully perpetration, it is still unclear how this relation unfolds over time. This study investigates whether victimization in childhood is a prospective risk factor for bully perpetration in early adolescence, and examines rumination as a socio-cognitive factor that may mediate this association. Participants included 553 third graders (43.2% boys; *M*_age_ = 9.85), with follow-up assessments when they were in fourth, seventh, and eighth grade. Results indicated that more frequent victimization in grades 3 and 4 was indirectly associated with bully perpetration in grade 8, through rumination in grade 7 about past victimization experiences in elementary school. This pattern remained regardless of whether the rumination elicited feelings of anger or sadness. Our findings demonstrate one pathway through which frequent victimization can lead to perpetration and underscore the important role of rumination in victims’ subsequent adjustment. Implications for future interventions are discussed.

Victimization is first and foremost associated with internalizing problems (e.g., depression, anxiety; Reijntjes et al., 2010); however, there is considerable evidence that victimization is also concurrently and prospectively associated with behavioral problems and externalizing symptoms for youth (e.g., Reijntjes et al., 2011). For example, early victimization (in childhood or early adolescence) is a risk factor for aggression (e.g., Reijntjes et al., 2011; Rusby, Forrester, Biglan, & Metzler, 2005; Schwartz, McFadyen-Ketchum, Dodge, Petit, & Bates, 1998). Moreover, there are some youth who are bullied yet also bully peers themselves (i.e., “bully-victims”; Yang & Salmivalli, 2013), and some evidence that a subset of youth who are victimized in childhood later turn to bully perpetration (Barker, Arseneault, Brengden, Fontaine, & Maughan, 2008; Haltigan & Vaillancourt, 2014; Walters, 2020a, 2020b; Walters & Espelage, 2018). Nevertheless, the mechanisms underlying the link between peer victimization and bully perpetration over time are still unclear. This is concerning, as bullying is associated with an assortment of psychosocial and behavioral difficulties for both victims and perpetrators (Nansel, Craig, Overpeck, Saluja, & Ruan, 2004). Furthermore, interventions targeting aggression and bullying are typically less effective in adolescence than childhood (Kärnä et al., 2013; Yeager, Fong, Lee, & Espelage, 2015). Therefore, it is important to identify pathways in which early victimization leads to bullying perpetration in adolescence to prevent cycles of aggression in the peer group.

Whereas past research has identified socio-cognitive factors (e.g., self-evaluations, rumination) as potential mediators between victimization and internalizing symptoms (e.g., Mathieson, Klimes-Dougan, & Crick, 2014; Troop-Gordon & Ladd, 2005), there is limited research examining socio-cognitive factors as mediators between victimization and aggression. Furthermore, the extant research has primarily focused on one type of social cognitive bias (i.e., hostile attribution bias) and only a handful of studies (e.g., Moon, Morash, & McCluskey, 2012; Walters, 2020b; Walters & Espelage, 2018) have examined pathways underlying the association between victimization and bullying perpetration, which is a specific type of aggression that may be associated with worse outcomes for victims (Felix, Sharkey, Green, Furlong, & Tanigawa, 2011; Ybarra, Espelage, & Mitchell, 2014). Besides hostile attribution bias, victimization is also associated with other maladaptive socio-cognitive factors, such as rumination (e.g., Monti, Rudolph, & Miernicki, 2017), that may play a role in whether victimization leads to perpetration. The current study builds on past research by examining whether the prospective association between victimization and bullying is mediated by rumination about past victimization. Furthermore, we will test whether there is a different pattern when rumination about past victimization elicits feelings of anger (i.e., angry rumination) compared to when rumination elicits feelings of sadness (i.e., sad rumination).

## Victimization as a Risk Factor for Future Bullying Perpetration

Bullying is distinct from other forms of aggression, both in terms of definition (e.g., Volk, Veenstra, & Espelage, 2017) as well as adjustment outcomes for perpetrators and victims (e.g.‚ Jia & Mikami, 2018; Ybarra et al., 2014). Bullying is typically defined as repeated aggression intended to harm the victim, with a power imbalance between the perpetrator and victim (Olweus, 1993). Victims of bullying specifically, as opposed to aggression more broadly, tend to report higher levels of internalizing symptoms as well as more social and academic difficulties (e.g., Hunter, Boyle, & Warden, 2007; Ybarra et al., 2014). As bullying is recognized internationally as a serious health concern due to its impact on victims’ mental and physical health (Reijntes et al., 2010), it is essential to understand potential mechanisms underlying bullying perpetration.

Moon, Morash, and McCluskey (2012) found that victimization longitudinally predicted bullying, and when examining trajectories of victimization and bullying, Barker and colleagues (2008) and Haltigan and Vaillancourt (2014) both found that a pathway from peer victimization to bullying was more likely than a pathway from bullying to victimization. Moreover, Walters and Espelage (2018) found an indirect effect of victimization on future bullying perpetration, mediated by hostility (i.e., thoughts and feelings of antagonism, resentment). Notably, a recent meta-analysis by Walters (2020a) found not only that bullying victimization and perpetration were strongly correlated, but that victimization was also a risk factor for future perpetration. Taken together, these studies support that victimization can lead to bullying perpetration over time.

## Victimization, Socio-Cognitive Difficulties, and Bullying

Although there is compelling evidence that victimization may be one factor that leads youth to bully, mechanisms underlying this association are still unclear. Victimization, and particularly frequent victimization, is associated with distinct social-cognitive processing (Rosen, Milich, & Harris, 2007). Consistent with the “victim schema model” (Rosen, Milich, & Harris, 2009), frequent victimization can lead to biased cognitive processing and difficulties with emotion regulation, which in turn can lead to aggressive responses to perceived threats. Whereas the victim schema model mostly focuses on how chronic victimization can impact their responses to distress “in the moment” (Rosen et al., 2009), it can also help explain why victimization could lead to elevated levels of bullying over time, such that chronic victimization hinders adaptive coping and stress responses. For example, youth who are frequently victimized tend to ruminate on their distress (e.g., Monti et al., 2017).

Rumination is a maladaptive, involuntary stress response that involves repetitive and intrusive cognitions and dwelling on distress (Nolen-Hoeksema et al., 2008), and rumination can elicit feelings of both sadness and anger (Peets, Hodges, & Salmivalli, 2013; Peled & Moretti, 2007, 2010). Social difficulties and other life stressors predict elevated rumination over time in adolescents (McLaughlin, Hatzenbuehler, & Hilt, 2009; Michl, McLaughlin, Shepherd, & Nolen-Hoeksema, 2013). Not only is victimization one stressor associated with high levels of rumination (e.g., Monti et al., 2017), but rumination can mediate the association between stressors and subsequent adjustment. Indeed, prior research indicates that rumination mediates the association between life stressors (including victimization) and internalizing symptoms (e.g., depression, anxiety; Feinstein, Bhatia, & Davilia, 2014; Mathieson et al., 2014; Michl, McLaughlin, Shepherd, & Nolen-Hoeksema, 2013). Moreover, there is burgeoning evidence that rumination (specifically brooding) may mediate the association between early life stress and externalizing symptoms (e.g., delinquent and aggressive behavior; LeMoult et al., 2019). As such, rumination may also play an important role in whether victimization predicts externalizing problems such as bullying perpetration; however, this has not yet been examined.

Victimization may lead to perpetration due to a desire for retaliation or to protect oneself from future victimization (e.g., Yeung & Leadbeater, 2007). This may be particularly true for youth at risk for developing a victim schema (i.e., chronic victims). Youth who have been frequently victimized are more likely to dwell on their past experiences of victimization (i.e., rumination) which in turn may predict their perpetration. Furthermore, Pedersen and colleagues (2011) found that provocation-focused rumination (i.e., brooding over a specific grievance or incident), but not self-focused rumination (i.e., focus on one’s own negative characteristics), predicted aggressive cognitions. Consistent with this finding, rumination about past victimization specifically (i.e., regarding a provocation) may be particularly relevant for youth’s bullying perpetration. Thus, we hypothesized that youth’s rumination on past victimization would mediate the prospective association between victimization and bullying.

Whether or not victimization is related to bullying through youth’s rumination on their past victimization may also be related to the feelings elicited by rumination (e.g., anger or sadness). Agnew’s (1985) general strain theory (GST), which focuses on the role of emotions in individual’s adjustment (rather than rumination specifically), supports the hypothesis that victimization would predict bullying, particularly if the victimization elicits angry rumination. GST explains how strains (i.e., stressful circumstances) can result in delinquency in individuals with ineffective coping strategies (Agnew, 1992). Specifically, GST posits that negative emotions (especially anger) explain why strains lead to delinquent behavior. In other words, when individuals experience a strain (e.g., victimization) it can lead to anger which in turn leads individuals to engage in deviant behavior.

The few studies that have examined potential mediators of the association between victimization and bullying specifically (rather than aggression more generally) have considered both emotional (e.g., anger) and cognitive (e.g., hostility) variables that are components of aggression (e.g., DeWall, Anderson, & Bushman, 2012). Surprisingly, the studies that examined anger as a mediator did not find it to be a significant mediator of the association between victimization and bullying (Moon et al., 2012; Walters, 2020b; Walters & Espelage, 2018). There are several reasons, however, to still expect angry rumination to mediate the association between victimization and bullying. First, angry rumination (i.e., dwelling on one’s anger) uniquely predicts aggression beyond the effects of general feelings of anger (Peled & Moretti, 2007). Second, past research examining emotional and cognitive components of variables that may mediate the association between victimization and perpetration suggest that the emotional component may not be sufficient on its own to be a mediator (e.g., Walters, 2020b; Walters & Espelage, 2018). Angry rumination includes an emotional component (anger), but also a strong cognitive component (rumination). Lastly, past studies have used items that assess trait anger rather than situational anger (Moon et al., 2012; Walter & Espelage, 2018). Situational anger refers to anger that arises from a specific stressor and has been shown to be a strong predictor of deviance (e.g., Mazerolle, Piquero, & Capowich, 2003). Thus, we posit that it is more likely for anger that arises from dwelling on past victimization (i.e., thinking about a specific stressor) to be a mediator of the association between victimization and perpetration than trait-based anger. As ruminating on past provocations is particularly relevant for aggressive cognitions (Pedersen et al., 2011), angry rumination about victimization specifically is likely to impact whether victimization leads to bullying over time. Insofar as victimization leads to perpetration due to a desire for retaliation or to protect oneself from future victimization (e.g., Yeung & Leadbeater, 2007), then it is more likely that youth’s angry rumination about their experiences being victimized specifically would be a mediator in the association between victimization and bullying.

Although ruminating on past victimization may elicit feelings of anger, it could also elicit feelings of sadness. In contrast to angry rumination, past research has identified that sad rumination is positively linked to depressive symptoms rather than aggression (e.g., Peled & Moretti, 2007). Youth who feel sad when ruminating on their past victimization may be more likely to withdraw or become depressed than to perpetrate bullying. Indeed, past research comparing youth who scored highly on different bullying participant roles found that victims, but not bullies, were more likely to say they would feel sad in response to a hypothetical provocation (Camodeca & Goossens, 2005). In the current study, we examined whether the feeling (i.e., anger or sadness) elicited by rumination on past victimization plays a role in the link between victimization and bullying. Specifically, we will also examine whether angry rumination is a unique mediator of this association, compared to sad rumination.

## The Current Study

The main objective of the current study was to demonstrate that rumination on past victimization mediates the association between early victimization and bullying over time. In addition, the current investigation aims to examine whether this mechanism is driven by angry rumination (compared to sad rumination). Despite evidence that victimization is a risk factor for bullying perpetration (e.g., Walters & Espelage, 2018), the mechanisms underlying the association between victimization and bullying (rather than aggression more generally) are not yet clear. In the current study, we focused on victimization in elementary school as early victimization can have lasting impact on adjustment in adolescence and even adulthood (e.g., McDougall & Vaillancourt, 2015). We examined rumination and bullying in early adolescence as rumination appears to become more prevalent in this developmental period (e.g., Jose & Brown, 2008) and because bullying interventions are typically less effective in adolescence (Kärnä et al., 2013).

Victimization is linked to maladaptive coping strategies and can also elicit negative emotions, both of which are associated with aggression (e.g., Peled & Moretti, 2007). As such, we expected frequent early victimization (i.e., in elementary school) to be positively associated with bullying perpetration in middle school. Furthermore, we expected more frequent victimization to positively predict rumination on past victimization, and for rumination in turn to predict bullying. Moreover, we expected angry rumination, rather than sad rumination, to mediate the link between victimization and bullying perpetration.

## Method

### Participants and Procedure

Participants were recruited from a cohort of third graders who participated in a large longitudinal project in Finland in Grades 3 and 4 (refer to Kärnä et al., 2011, 2013 for a more detailed description of the project). A follow-up study with a subset of the sample was conducted when the students were in Grade 7 and 8. The original cohort included 2729 students, but only the ones from Finnish-language schools (N = 65 schools) were approached for the follow-up study. Out of the 65 school principals contacted to assist with recruiting students for the follow-up, 57 principals agreed to the data collection. In total, 821 students (41% boys) received positive parental consent and provided individual assent forms to participate in the study. As the goal of the current study was to examine whether rumination on *past victimization* mediated the association between victimization in elementary school and bullying in middle school, we limited our sample to youth who had been victimized – even if infrequently – in elementary school. Thus, for the analyses in the current study, we included 553 participants (43.2% boys; *M*_age_ = 9.85, *SD* = 0.71) who reported at least some victimization in Grade 3 or Grade 4 (see Measures below for more information). Of these 553 participants, 484 participants provided information regarding bullying perpetration in Grade 8. Most participants (97.6%) were native Finns (i.e., Caucasian). Information regarding family socioeconomic status and ethnic background was not collected, due to the socioeconomic and ethnic/racial homogeneity in Finland, especially at the time of data collection.

In Grade 3 and Grade 4, students completed online questionnaires during school at three time points (at the end of Grade 3, the middle of Grade 4, at the end of Grade 4). The administration of the questionnaires was supervised by teachers who received detailed instructions regarding the procedure two weeks prior to data collection. Students were reassured of the confidentiality of their answers. When the students were in Grade 7 and 8, consenting students completed an online questionnaire in their free time or could complete the questionnaire via pen-and-paper. This procedure was in concordance with the ethical guidelines of the University of Turku.

**Fig. 1:**
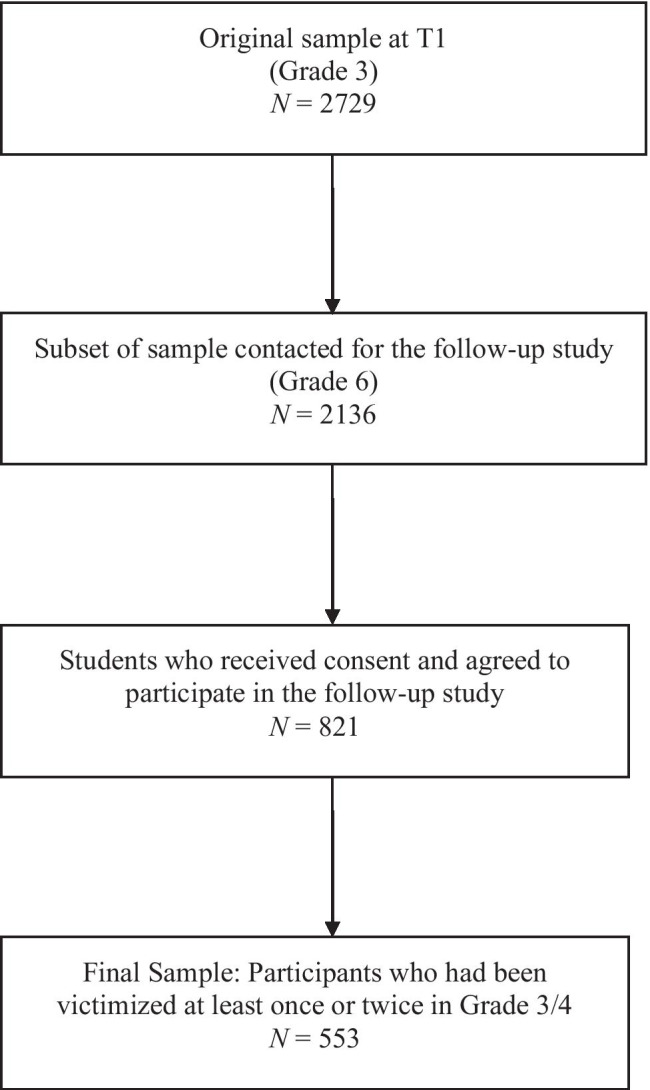
Flowchart of included participants

We conducted exploratory analyses to test whether the participants in the follow-up study (n = 821) significantly differed from the sample in the original study (see Fig. 1 for a flowchart of included participants). Participants in the follow-up study reported lower levels of victimization at the end of Grade 3 (*t* = 2.09, *p* = 0.04) and the middle of Grade 4 (*t* = 2.53, *p* = 0.01), and lower levels of bullying at the end of Grade 3 (*t* = 3.17, *p* = 0.002). Participants in the follow-up study did not differ on victimization at the end of Grade 4, bullying at any time point in Grade 4, or on other adjustment variables (depressive symptoms, anxiety). A logistic regression analysis was run to examine whether victimization or bullying in Grades 3 and 4 predicted whether students participated in the follow-up study. The model was not statistically significant, χ^2^(6) = 9.13, *p* = 0.16, Nagelkerke *R*^2^ = 0.01. Victimization and bullying at any time point were not related to participating in the follow-up study.

## Measures

**Victimization (Grades 3 and 4).** Participants completed the Olweus Bully/Victim questionnaire (OBVQ-R, Olweus, 1996) in at the end of Grade 3, the middle of Grade 4, and the end of Grade 4. Students were given the definition of bullying, then asked “How often have you been bullied by others at school in the last two months?” Youth responded on a five-point scale (0 = “Not at all”, 1 = “Once or twice”, 2 = “Two or three times a month”, 3 = “Every week”, 4 = “Several times a week”). This item was only used to identify the final sample, which included any participant who indicated being victimized at least “once or twice” at any of the three time points.

At the end of Grade 3 and Grade 4, participants were also asked to complete a 10-item scale assessing their experiences of direct (e.g., being called names; being hit or pushed) and indirect forms of victimization (e.g., social exclusion), along with their experiences of victimization with racist and sexual content and cybervictimization, using the same five-point Likert scale described above. Participants’ responses were averaged across the items and across Grades 3 and 4. The scales had adequate internal consistency with Cronbach’s α-s of 0.80 (Grade 3), 0.82 (Grade 4), and 0.85 (Grades 3 and 4 averaged). In the current study, we used the averaged victimization score for our analyses, as we were interested on the total amount of victimization experienced during the two elementary school years, rather than any specific form of victimization or any particular time point.

Peer-reported victimization was also assessed in Grades 3 and 4, using 3 items from the Participant Role Questionnaire (PRQ; Salmivalli & Voeten, 2004; e.g., “s/he is called names and made fun of”). Students could nominate an unlimited number of classmates for each item. The received nominations for each participant were summed and divided by the number of possible nominators within each classroom to form a proportion score. The peer-reported victimization score at each time point was created by averaging across the 3 items. We then averaged the scores for end of Grade 3, beginning of Grade 4, and end of Grade 4 to form the final peer-reported victimization score.

**Bullying (Grades 3 and 8).** As part of the Olweus Bully/Victim questionnaire (OBVQ-R, Olweus, 1996), before participants reported their bullying behaviors, they were provided with a definition of bullying that captures three main components of bullying (i.e., intention to harm, repetitive nature of bullying, and a power imbalance between the perpetrator and victim); the definition also differentiated between bullying and playful teasing. Participants then indicated “How often have you bullied others at school in the last two months?” The participants answered this question using a five-point scale (0 = “Not at all”, 1 = “Once or twice”, 2 = “Two or three times a month”, 3 = “Every week”, 4 = “Several times a week”) in Grades 3 and 8. This item has been shown to demonstrate adequate construct validity and psychometric properties (Solberg & Olweus, 2003). Bullying was log transformed due to the non-normal distribution of bullying in both grades.

**General, angry, & sad rumination (Grade 7).** Students completed a modified rumination scale by McCullough and colleagues (2007) to capture the extent to which the participants dwell on past victimization experiences. Participants only completed this scale if they recalled past experiences of being victimized. To assess whether students recalled being bullied in elementary school, they were provided the definition of bullying again and asked if they had ever been bullied in Grades 1 to 6. Participants responded using a three-point scale (“not at all”, “to some extent”, “all the time”). Participants who indicated that they had been bullied at least “to some extent” in Grades 1 to 6 were then asked to fill out the 8-item rumination scale (e.g., “I can’t stop thinking about what the bullies did to me”).

To differentiate between angry and sad rumination, two additional questions were asked after each of the items on the rumination scale (“If/when this happens, to what extent do you feel: (a) sadness and distress; (b) anger and rage?”). Participants reported the degree to which the rumination elicited anger (angry rumination) and sadness (sad rumination) using a four-point scale (0 = “not true of me at all”/ “not at all”…. 3 = “completely true of me”/ “a lot”). For individuals who responded that they had not experienced any victimization in Grades 1 to 6, we imputed the value of zero for all rumination items. The internal consistency of the rumination items was high, with Cronbach’s α-s of 0.94 for general rumination, 0.94 for sad rumination, and 0.95 for angry rumination.

## Analytic Plan

To test the direct and indirect effects of early victimization (Grade 3/4) on bullying in Grade 8, we conducted SEM in R using the laavan package (Rosseel, 2012). We evaluated model fit based on conventional measures of fit: nonsignificant χ^2^ value, comparative fit index (CFI) > 0.9, root mean square of approximation (RMSEA) < 0.08, and standardized root mean square residual (SRMR) close to zero (Kline, 2004). We first conducted models using Full Information Maximum-Likelihood estimation (FIML) with robust standard errors (MLR) to address missing data. Of the final sample, those with any missing data (n = 31) did not significantly differ on any of the predictor variables compared to participants with complete data. Next, to formally test for significant indirect and direct effects in the mediation models, we used bias-corrected bootstrapped 95% confidence intervals, with 5,000 bootstrap draws (Preacher & Hayes, 2008) which uses Maximum-Likelihood estimation (ML). If the confidence interval did not include zero, then the effect was considered significant. As the confidence intervals were rounded to two decimal points, it is possible for the lower or upper bound to be rounded to “0.00”. In these cases, if the lower and upper bound are both positive or both negative, then this indicates that the confidence interval did not contain zero.

Two models were conducted to test our hypotheses. In Model 1, we tested general rumination in Grade 7 as a mediator of the association between victimization in Grade 3/4 and bullying in Grade 8. In Model 2, we tested angry and sad rumination separately as potential mediators, while controlling for the correlation between angry and sad rumination. Gender and bullying in Grade 3 was controlled for in both models.

## Results

### Descriptive Statistics

Means, standard deviations, and correlations among study variables are depicted in Table 1. Girls reported more general rumination, as well as sad rumination, than boys; however, there were no gender differences in angry rumination. Boys were more likely than girls to report bullying others in grades 3 and 8. There were no significant gender differences in elementary school victimization. Victimization across grades 3 and 4 was positively associated with bullying in grade 3 and both types of rumination in Grade 7 (*rs* ranging from 0.20 to 0.31). General rumination was positively related to bullying in grade 8 (*r* = 0.13).

**Table 1:** Correlations, means, and independent sample *t*-tests

	1	2	3	4	5	*M* (SD)	*M* (SD)_boys_	*M* (SD)_girls_	*t*
1. Victimization G3/G4	–					0.58 (0.42)	0.60 (0.43)	0.56 (0.41)	-1.04
2. Rumination G7	0.27^***^	–				0.42 (0.63)	0.35 (0.52)	0.48 (0.70)	2.52^*^
3. Angry rumination G7	0.20^***^	0.71^***^	–			0.41 (0.69)	0.37 (0.65)	0.43 (0.72)	0.99
4. Sad rumination G7	0.26^***^	0.87^***^	0.77^***^	–		0.39 (0.65)	0.31 (0.56)	0.45 (0.70)	2.55^*^
5. Bullying G3	0.31^***^	0.07	0.02	0.02	–	0.50 (0.72)	0.63 (0.74)	0.40 (0.69)	-3.49^*^^**^
6. Bullying G8	0.06	0.13^**^	0.08	0.09	0.17^***^	0.26 (0.61)	0.38 (0.76)	0.17 (0.46)	-3.31^**^

*G3* Grade 3, *G4 *Grade 4, *G7* Grade 7, *G8 *Grade 8

*p < 0.05; **p < 0.01; ***p < 0.001

### Model 1: From Victimization to General Rumination to Bullying

**Fig. 2:**
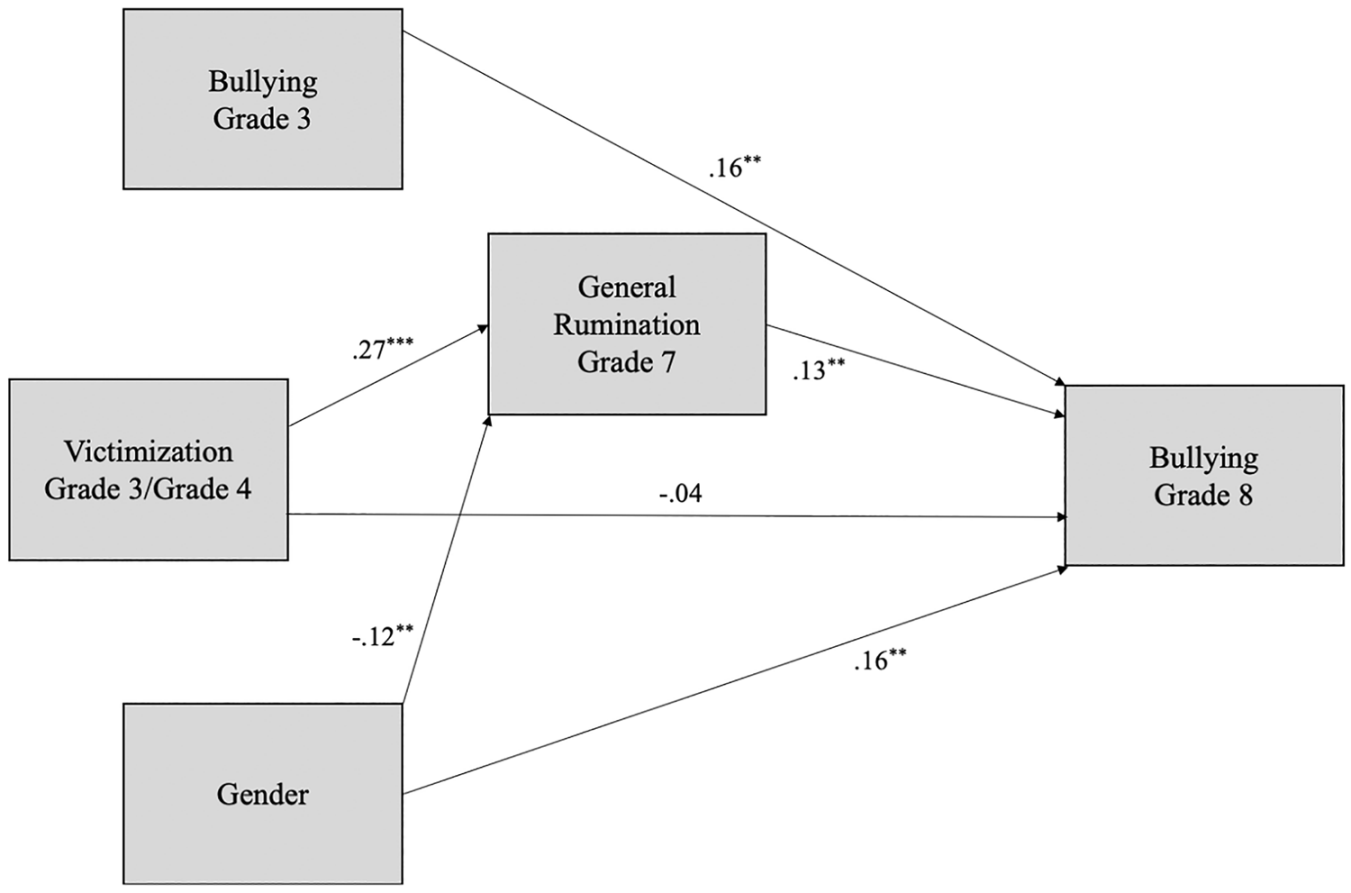
General rumination in Grade 7 as a mediator between victimization in Grade 3/4 and bullying in Grade 8. Standardized coefficients are presented. For ease of presentation, correlations among predictors are not shown. The indirect path from victimization to bullying mediated by general rumination is significant. **p < 0.01, ***p < 0.01

In Model 1, we tested whether the association between victimization and bullying was mediated by general rumination (Fig. 2). The model had adequate fit, χ^2^(1) = 0.03, *p* = 0.86, CFI = 1.00, RMSEA = 0.00 (90% CI [0.00, 0.07], SRMR = 0.00. Victimization in Grades 3/4 was positively associated with rumination in Grade 7 (β = 0.27), which in turn was positively associated with being a bully in Grade 8 (β = 0.13), even when controlling for the stability of bullying from Grade 3 to Grade 8 (β = 0.16). There was a significant indirect effect (0.01) from victimization to bullying, mediated by rumination, 95% CI [0.00, 0.02]. The direct effect (-0.01) of victimization on bullying was not significant, 95% CI [-0.05, 0.03]. To explore whether the pattern of results was due to common method bias, we also tested this model using peer-reported victimization in Grade 3/4. Again, there was a significant indirect effect from victimization to bullying (0.12), mediated by general rumination, 95% CI [0.05, 0.24], whereas the direct path from peer-reported victimization to bullying was not significant, 95% CI [-0.39, 0.04].

### Model 2: From Victimization to Angry Rumination to Bullying

**Fig. 3:**
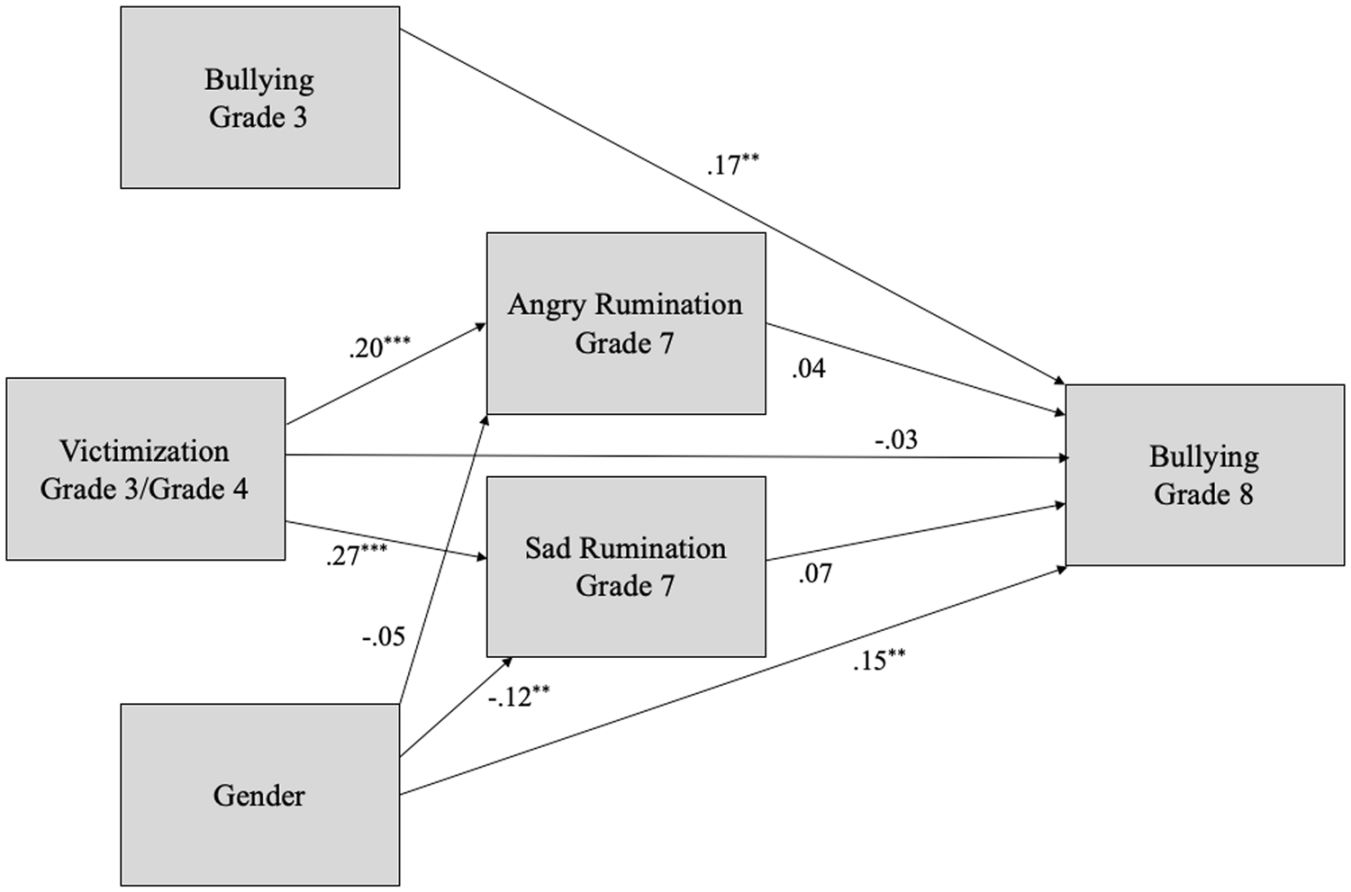
Angry and sad rumination in Grade 7 as mediators between victimization in Grade 3/4 and bullying in Grade 8. Standardized coefficients are presented. For ease of presentation, correlations among predictors and the residual correlation between angry and sad rumination are not shown. The total indirect effect from victimization to bullying is significant. *p < 0.05, **p < 0.01, ***p < 0.001

In Model 2, we tested whether angry rumination specifically mediated the associated between victimization and bullying, compared to sad rumination (Fig. 3). Model 2 also had adequate fit, χ^2^(2) = 0.69, *p* = 0.71, CFI = 1.00, RMSEA = 0.00 (90% CI [0.00 – 0.06], SRMR = 0.01. Victimization was positively related to both angry (β = 0.20) and sad (β = 0.27) rumination. Neither the path from angry rumination nor sad rumination in Grade 7 was significantly associated with bullying in Grade 8. However, the total indirect effect (0.01) from victimization to bullying via the mediators was significant, 95% CI [0.00, 0.02]. In contrast, the direct path (-0.01) from Grade 3/4 victimization to Grade 8 bullying was not significant, 95% CI [-0.05, 0.03]. As before, we tested this model again using peer-reported victimization in Grade 3/4. There was also still a significant total indirect effect (0.08) from victimization to bullying, 95% CI [0.02, 0.19]. The direct path from peer-reported victimization to bullying was not significant, 95% CI [-0.36, 0.09].

## Additional Exploratory Analyses

Given that the total indirect effect in Model 2 was significant but the individual indirect paths via angry rumination and sad rumination were not significant, we conducted exploratory analyses with angry and sad rumination in separate models. The model with only angry rumination as a mediator fit well, χ^2^(1) = 0.54, *p* = 0.47, CFI = 1.00, RMSEA = 0.00 (90% CI [0.00, 0.11], SRMR = 0.01. There was a significant indirect effect (0.01) from self-reported victimization in Grade 3/4 to bullying in Grade 8, mediated by angry rumination in Grade 7, 95% CI [0.00, 0.02]. The model with only sad rumination also had adequate fit, χ^2^(1) = 0.52, *p* = 0.47, CFI = 1.00, RMSEA = 0.00 (90% CI [0.00,0.00], SRMR = 0.01. The indirect effect (0.01) from self-reported victimization to bullying, mediated by sad rumination was also significant, 95% CI [0.00, 0.02]. The direct path from self-reported victimization to bullying was not significant in either model. We found the same pattern of results when using peer-reported victimization.

## Discussion

Given the deleterious consequences of bullying on victims’ mental and physical health (e.g., Reijntjes et al., 2010), it is essential to understand factors that predict bully perpetration. In the current investigation, we focused on one possible (and understudied) pathway that may predict bullying: youth’s past experiences of being victimized and how they coped with this experience. Specifically, we examined whether victimization in elementary school (Grades 3 and 4) was longitudinally associated with bullying in middle school (Grade 8), and whether this association was mediated by youth’s rumination on their past victimization. In a sample of youth who all had reported at least some victimization experiences in elementary school, those who had been victimized more frequently were more likely to bully others in middle school, and this association was mediated by rumination on past victimization. Although a substantial amount of research indicates that some youth are both perpetrators and victims of bullying concurrently (e.g., Yang & Salmivalli, 2013), this study adds to research demonstrating that victimization can actually lead to bullying perpetration over time (e.g., Barker et al., 2008; Haltigan & Vaillancourt, 2014; Moon et al., 2012; Walters, 2020a; Walters & Espelage, 2018). Moreover, consistent with past research (e.g., Troop-Gordon & Ladd, 2005), this study underscores that (mal)adaptive socio-cognitive processes play a crucial role in the extent to which victimization leads to negative outcomes.

## Victimization, Rumination, and Bullying

While frequent early victimization was prospectively associated with bullying in middle school, it is important to remember that not all youth who are victimized eventually bully others. Indeed, past research examining joint trajectories of victimization and bullying over time (Barker et al., 2008; Haltigan & Vaillancourt, 2014) found that only a small percentage (≤ 6%) of all youth were in the “victim-to-bully” group. As not all youth who are victimized become aggressive, it is crucial to understand potential mechanisms that underlie when victimization does predict perpetrating bullying over time.

As hypothesized, rumination on past victimization mediated the prospective association between victimization and bullying. Elevated frequencies of victimization in elementary school was associated with increased rumination in middle school. Moreover, for youth who were victimized more frequently, rumination on victimization in turn was associated with bullying. When differentiating between angry and sad rumination, we found that high levels of victimization was associated with both angry and sad rumination. That is, youth who had experienced more frequent victimization were especially likely to experience rumination that elicited both anger and sadness.

Although we expected angry rumination, rather than sad rumination, to be a unique mediator of the association between victimization and bullying, neither emerged as a significant mediator in the model with both angry and sad rumination. Nevertheless, the overall indirect effect (which included both angry and sad rumination as mediators) was significant. Additional exploratory analyses examining angry and sad rumination as mediators in separate models indicated that, as expected, angry rumination mediated the association between victimization and bullying. However, contrary to our expectations, sad rumination was also a significant mediator. In other words, for victimized youth, becoming angry or sad when dwelling on past experiences of victimization makes it more likely to show increases in perpetration. There are several possible explanations for this pattern of findings. For example, youth who tend to ruminate about their victimization may feel justified to bully others because of their past experiences regardless of the specific negative emotion elicited (i.e., anger or sadness). Our findings are consistent with general strain theory (GST), which purports that ineffective coping strategies and negative emotions in general explain why stressors (e.g., victimization) lead to deviant behavior. Alternatively, there may be differential effects between angry and sad rumination that we were unable to detect due to how angry and sad rumination were measured. Indeed, angry and sad rumination were highly correlated and youth may feel simultaneously angry and sad when ruminating on their past victimization experiences.

Whereas past research has examined the role of anger and/or hostility in the association between victimization and bullying (Moon et al., 2012; Walters & Espelage, 2018; Walters, 2020b), the current study, to our knowledge, is the first to demonstrate that rumination specifically about past experiences of victimization mediates the link between victimization and perpetration. Taken together, our results demonstrate that rumination is one pathway in which victimization influences subsequent bullying, but that there may be other pathways through which victimization leads to bullying. Future research could also utilize person-centered analyses to identify different groups of victimized youth and their rumination or other coping strategies and compare their subsequent adjustment.

Whereas some school-based interventions have focused on the role of coping strategies in decreasing aggression in adolescence, many interventions target coping skills more generally (e.g., cognitive distortions, problem-solving strategies; Powell et al., 2011) and do not necessarily focus on rumination. Furthermore, while the findings are mixed as to whether coping skills interventions help reduce aggressive responses to provocations or social rejection (see Yeager, Trzesniewski, & Dweck, 2013), there are key differences between rumination and other maladaptive socio-cognitive processes. For example, rumination is associated with residual difficulties after treatment (e.g., lingering subsyndromal symptoms), as it can be less responsive to treatment or interventions (e.g., Watkins et al., 2007). Some programs that focus on learning anger control skills and cognitive-behavioral therapy (e.g., socially appropriate reactions, regulating anger expression; Powell et al., 2011; Sukhodolsky, Smith, McCauley, Ibrahim, & Piasecka, 2016) may help youth manage their anger expression in the moment but may not necessarily help a child avoid ruminating on past transgressions against them. In other words, a child who has been victimized could use coping skills and constructive problem-solving skills to avoid immediately lashing out when victimized, but could still continue to have intrusive thoughts about their past victimization, particularly when they have been victimized frequently. That is, it is likely harder for youth who are repeatedly victimized to avoid repetitive or intrusive thoughts about being victimized. Although some strategies, such as mindfulness, are effective in reducing rumination in adolescence (e.g., Ames, Richardson, Payne, Smith, & Leigh, 2014), future research is needed to examine interventions that could help adolescents minimize rumination after on-going victimization.

## Strengths, Limitations, and Future Directions

The current investigation adds to longitudinal research examining the association between victimization and bullying perpetration and is the first to identify rumination as a key socio-cognitive factor underlying this link. Still, there are limitations of the current study that should be acknowledged.

The current study aimed to examine the distinct mediating effects of angry and sad rumination; however, there was considerable overlap in angry and sad rumination. These forms of rumination were highly correlated (*r* = 0.77) and we found similar pattern of findings for both types of rumination. Therefore, our findings may reflect the impact of rumination in general on the link between victimization and bullying, rather than the specific emotions elicited by rumination. Alternatively, it is also possible that any negative emotions (i.e., both anger and sadness) elicited by rumination mediate the association between victimization and bullying. Given that our findings are inconsistent with past research that has demonstrated differential effects of angry and sad rumination (e.g., Peled & Moretti, 2007, 2010), future research is needed to better understand potential differences in angry and sad rumination about past victimization. For example, person-centered analyses could identify whether there are subtypes of victimized youth who primarily feel angry, primarily feel sad, or typically feel both angry and sad when ruminating on past victimization, and how that predicts adjustment. Moreover, even if both angry and sad rumination predict bullying perpetration, it is possible that they may be differentially related to other outcomes.

Furthermore, bullying was assessed with the global bullying item of the self-report Olweus Bully/Victim questionnaire (OBVQ-R, Olweus, 1996). While the current study provides novel insight to the link between victimization and bully perpetration over time, future research would benefit from testing the proposed mediational model with a measure that assesses multiple forms of bullying and/or with peer-nominated bullying. However, the current study did include both self- and peer- reports of victimization, and sensitivity analyses indicated that the proposed mediational models were still significant when using peer-reported victimization. Moreover, whereas the current study focused on any experience of victimization in elementary school, youth whose victimization remained stable across all of elementary school may differ from youth who were victimized at only one time point. Future research should consider other characteristics of victimization (e.g., stability of victimization, whether the child is victimized by many peers or just one classmate) that may also be related to the proposed mechanism.

Although the longitudinal design is a strength of the current study, it is important to consider the amount of time between each wave. For example, causal effects (mediation) typically occur within a shorter time frame than is often captured by longitudinal studies of peer relations (e.g., data points separated by years; de Castro et al., 2015). In the current study, there was a 3-year gap between the predictor (victimization) and mediator (rumination). Thus, it is possible that during this time there were other confounding variables that influenced the association. However, we believe that the focus on rumination about past victimization specifically (rather than rumination more generally) helps mitigate this concern. In addition, as the current study was a follow-up to a larger school-based survey and took place years later outside of the school context, we were not able to retain the full original sample. Nevertheless, our final sample was generally representative of the original sample, and key variables (i.e., victimization and bullying) did not significantly predict whether students participated in the follow-up study.

## Conclusions

Early victimization has lasting consequences for victimized youth's  psychosocial adjustment throughout development (McDougall & Vailliancourt, 2015). Despite substantial evidence that victimization is a risk factor for bullying (e.g., Walters, 2020a), the mechanisms underlying this association are still unclear. The current study expands on past research by demonstrating that rumination, a socio-cognitive process that becomes more prevalent in early adolescence (Jose & Brown, 2008), is one factor that underlies the prospective association between victimization and perpetration. Our findings highlight that interventions focused on maladaptive coping strategies should target rumination. Moreover, while the current study focused on perpetration, rumination on past victimization may also be associated with other adjustment outcomes. For example, rumination on victimization may influence other aspects of peer relationships or adjustment at school (e.g., loneliness, sense of belonging, academic performance, feelings of safety).
